# Bone Morphogenetic Proteins Stimulate Mammary Fibroblasts to Promote Mammary Carcinoma Cell Invasion

**DOI:** 10.1371/journal.pone.0067533

**Published:** 2013-06-28

**Authors:** Philip Owens, Hannah Polikowsky, Michael W. Pickup, Agnieszka E. Gorska, Bojana Jovanovic, Aubie K. Shaw, Sergey V. Novitskiy, Charles C. Hong, Harold L. Moses

**Affiliations:** 1 Department of Cancer Biology and Vanderbilt-Ingram Cancer Center, Vanderbilt University, Nashville, Tennessee, United States of America; 2 Research Medicine, Veterans Affairs Tennessee Valley Helathcare System, Nashville, Tennessee, United States of America; 3 Departments of Medicine, Pharmacology, and Cell and Developmental Biology, Vanderbilt University, Nashville, Tennessee, United States of America; Seoul National University, Republic of Korea

## Abstract

Bone Morphogenetic Proteins (BMPs) are secreted cytokines that are part of the Transforming Growth Factor β (TGFβ) superfamily. BMPs have been shown to be highly expressed in human breast cancers, and loss of BMP signaling in mammary carcinomas has been shown to accelerate metastases. Interestingly, other work has indicated that stimulation of dermal fibroblasts with BMP can enhance secretion of pro-tumorigenic factors. Furthermore, treatment of carcinoma-associated fibroblasts (CAFs) derived from a mouse prostate carcinoma with BMP4 was shown to stimulate angiogenesis. We sought to determine the effect of BMP treatment on mammary fibroblasts. A large number of secreted pro-inflammatory cytokines and matrix-metallo proteases (MMPs) were found to be upregulated in response to BMP4 treatment. Fibroblasts that were stimulated with BMP4 were found to enhance mammary carcinoma cell invasion, and these effects were inhibited by a BMP receptor kinase antagonist. Treatment with BMP in turn elevated pro-tumorigenic secreted factors such as IL-6 and MMP-3. These experiments demonstrate that BMP may stimulate tumor progression within the tumor microenvironment.

## Introduction

Within the family of Transforming Growth Factor β (TGFβ) are Bone Morphogenetic Proteins (BMPs), which can induce differentiation, growth arrest, apoptosis and many other distinct responses [1], [2]. There are more than 20 BMP ligands, which are secreted and processed as homo and/or heterodimers. Secreted soluble antagonists, including Noggin, Chordin, and Gremlin can inhibit BMPs [3]. When ligands bind to either type I or type II serine/threonine kinase receptors, they phosphorylate Smad1, Smad5 and/or Smad8 [4], [5]. These Smads next translocate in combination with Smad4 to the nucleus and regulate transcription of key target genes. One key element to the signaling behavior of BMP and TGFβ in general is the ability to induce negative feedback. Transcriptional targets as well as proteins at every step of activation are induced to self-limit BMP activity, which makes for a finely tuned system. Activation of canonical BMP signaling at the protein level is measured by phosphorylation of Smads 1, 5 and 8 [2]. While measurement of a BMP transcriptional response is measured by target genes (Id1, Smad6 and Smad7), inhibitory Smad proteins (Smad6 and Smad7) are some of the most prominent targets of active BMP signaling [2].

Fibroblasts in the tumor microenvironment have been shown to be promoters of tumor progression and metastasis [6], [7], [8], [9], [10]. Fibroblasts in breast cancer can support tumor growth by several direct and indirect mechanisms. First, fibroblasts can directly act upon tumor cells to stimulate growth and evade apoptosis. Second, fibroblasts can regulate the extracellular matrix or physical structure of the tumor microenvironment by enzymatically modulating Extra-Cellular Matrix (ECM) components such as collagen, fibronectin, and components of the basal lamina. Regulation of the stiffness and physical structure of the ECM can promote tumor cell growth and metastatic dissemination [11]. Third, fibroblasts can regulate the other stromal cell populations or induce their recruitment. Fibroblasts can also regulate angiogenesis and help to stimulate new vessel growth to support tumors [12]. Our laboratory has previously shown that loss of TGFβ signaling in fibroblasts can recruit inflammatory cells, which promote mammary tumor progression and metastasis [13], [14], [15]. This dynamic of TGFβ in tumor suppression and progression has led us to investigate BMP effects, which has also shown conflicting roles as both tumor suppressor and promoter.

BMP signaling has recently shown tumor suppressive phenotypes in mammary carcinomas, whereby disruption of BMP signaling in the epithelial compartment accelerates tumor progression [16]. Interestingly, breast cancers are characterized by an increase in BMP4 and BMP7 ligands [17]. We were interested in determining whether this increase may have distinct effects on cells in the tumor stromal microenvironment, which can have paracrine effects on carcinoma cells. Recently, it was discovered that fibroblasts derived from mouse prostate tumors stimulated by BMPs can increase angiogenesis via the upregulation of the chemokine SDF1α/CXCL12 [12]. This finding was supported by earlier work demonstrating that BMPs were playing active roles in the promotion of prostate tumorigenesis and subsequently bone metastases [18], [19]. Another clue that BMPs could have a unique function in fibroblasts came from a recent study demonstrating distinct transcriptional responses in human keratinocytes when compared to their underlying dermal fibroblasts. Intriguingly, a list of BMP induced genes contained many factors that have been demonstrated to promote cancer progression, such as IL-11, CTGF, and ADAM12 [20]. Here we demonstrate a tumor promotion role for BMPs in mammary fibroblasts by increasing secretion of inflammatory cytokines and matrix-metallo proteases (MMPs).

## Materials and Methods

### Cell Culture, Recombinant Proteins, Proliferation, Viability/Toxicity and Invasion Assays

Isolation of mammary mouse fibroblasts was performed as previously described [13]. Virgin female mice were euthanized and mammary glands were removed, minced and placed in DMEM media (Gibco) supplemented with triple antibiotic (Gibco) and 10% fetal bovine serum (Atlanta Biologicals). All mouse work was done according to the requirements of Vanderbilt University IUCAC protocol #M/07/331 regulating animal welfare to ameliorate any unnecessary suffering. Animals were sacrificed using CO2 asphyxiation. Cells were passaged through crisis and validated for fibroblast markers and morphology (Fig. S1). Establishment of human fibroblast cell lines from fresh tumor and normal tissues have been obtained from de-identified female patients. Vanderbilt University Institutional Review Board (application IRB# 080603 “TGFβ in mammary development and tumorigenesis”) was reviewed and determined that the study does not qualify as “human subject” research per §46.102(f)(2). Tissue was obtained from the pathology lab before being discarded. No identifiers were included and there was no contact with donor. Written permission from the IRB approved the research independent of an ethics committee. Tissue was washed in 15 ml of sterile DMEM F12 containing fungizone, gentamicin and penicillin streptomycin. Tissue was than transferred into a petri dish containing digestion media (DMEM 10% FCS, fungizone, gentamicin, penicillin streptomycin, collagenase, hyaluronidase) where it was finely minced using sterile scalpel and scissors. Minced tissue was then transferred to sterile 50 ml conical tube containing additional 5 ml of fresh digestion media. Minced tissue and 15 ml of digestion media was place in 37 degrees water bath shaker for 4 hrs. After 4 hrs of shaking/vortexing, digested tissue was centrifuged at 1000 rpm for 5 minutes. The remaining pellet was washed multiple times with DMEM F12∶10% FCS, fungizone, penicillin streptomycin, and gentamicin. Human fibroblasts from normal (NAF) and breast cancer (CAF) were initially grown in MCDB 131 media (Gibco) supplemented with 10% FBS, Insulin-Transferrin-Selenium, Non-Essential Amino Acids, L-glutamine, Aminomax basal medium and Aminomax C100 Supplement (all from Gibco). Once established, these cells were weaned into DMEM media containing triple antibiotic and 10% fetal bovine serum. Recombinant BMP-4 and Noggin was obtained from R&D Systems; mouse protein was used for mouse cells, and human protein used for human cells. DMH1 was synthesized and resuspended in DMSO as previously described [21]. Proliferation was assessed by incorporation of tritiated-Thymidine as previously described [16], which was added 22 hours after BMP4 treatment for two hours prior to fixation and collection for measurements. Viability and Toxicity of DMH1 titer in fibroblasts was determined using MultiTox-Glo Multiplex Cytotoxicity Assay (Promega) in a 96-well plate following manufacturers guidelines. Invasion assays were performed using 8 µM pore Matrigel coated 24 well plate invasion system (BD). Mouse mammary fibroblasts (C57BL6) were first seeded into the underlying well at a density of 5×10^4^ and allowed to grow for 24 hours prior to treatments. 2.5×10^4^ C57BL6 mouse derived MMTV-PyVmT cells were used and allowed to invade for 18 hours. Human mammary fibroblasts derived from reduction mammoplasty tissue were seeded at a density of 5×10^4^ and allowed to grow for 48 hours prior to treatments. 1×10^5^ MCF7 cells per 24 well were allowed to invade for 48 hours. Invasion chamber were stained overnight in Hematoxylin, removed and mounted onto microscope slides and quantified.

### RNA Isolation, cDNA Synthesis and Real-time PCR

RNA was purified with RNeasy Mini kit including DNaseI treatment plus QIAshredder columns (Qiagen). cDNA synthesis performed using VILO cDNA kit (Life Technologies). SYBR green master mix is LuminoCt (Sigma). Primers designed using NCBI-GENE-‘Pick Primers’ (Table S1), melting curves inspected after every run performed on BioRad CFX96 real time cyclers. All primers were optimized for 60 degree annealing and two-step cycling was performed from 95 degrees (10s) to 60 degrees (30s) for 40 cycles. GAPDH was used to calculate normalized fold change.

### Cytokine Antibody Array and RT^2^ Profiler PCR Arrays

Membrane bound antibody arrays Mouse Cytokines 3,4,5 Cat# AAM-CYT-2000-4 were obtained from RayBio and incubated with conditioned medium. Exposure was performed with ECL plus, images were scanned at high resolution and analyzed for intensity using NIH image J software. RT-PCR focused arrays were purchased from SABiosciences/Qiagen and performed as instructed by manufacturers protocol, including RNA purification, cDNA synthesis, real time instrumentation protocol and analyzed via web based tools provided (http://www.sabiosciences.com/pcrarraydataanalysis.php). Specifically, the following arrays were used: ECM (cat#PAMM-013) and TGFβ (cat#PAMM-035).

### Protein Isolation, Western Blot and ELISA

Total protein was isolated using Complete LysisM Buffer (Roche) and centrifuged to remove debris. Protein concentration was determined by microplate DC Bradford assay (BioRad). Protein was diluted to equal concentrations and equally loaded on 10% polyacrylamide gels and transferred to nitrocellulose membranes. Blots were incubated overnight with primary antibodies at the following concentrations: Smad1 (cat# D59D7) 1∶1000, Smad5 (cat# 9517), pSmad1/5 (cat# 9516), pSmad1/5/8 (cat# 9511), Smad6 (cat# 9519) 1∶1000, Actin (cat #sc-7210 ) 1∶4,000, ID1 (cat# sc-488) 1∶200. Actin and ID1 antibodies purchased from Santa Cruz, all Smad antibodies were purchased from Cell Signaling. ELISA for human IL-6 (bioLegend) Cat # 430504 Human IL-6 ELISA MAX™ Deluxe. ELISA for human MMP3 (AnaSpec) Cat #72103 SensoLyte® MMP - 3 ELISA Kit *Colorimetric* were performed following manufacturers guidelines.

### Immunofluorescence Staining

Immunofluorescence staining was performed by diluting all primary and secondary antibodies in 12%BSA. The following antibodies and dilutions were used: pSmad 1/5/8 (Cell Signaling) 1∶200, aSMA (Sigma) 1∶500, Vimentin (Covance) 1∶500 Fsp-1 (eBiosciences) 1∶200 PDGFRa (eBiosciences) 1∶200. Secondary antibodies were all goat derived highly cross-adsorbed and used at 1∶200. Slides were mounted in SlowFade +DAPI (Molecular Probes/Invitrogen).

## Results

### BMP Response in Mouse Mammary Fibroblasts

**Figure 1:**
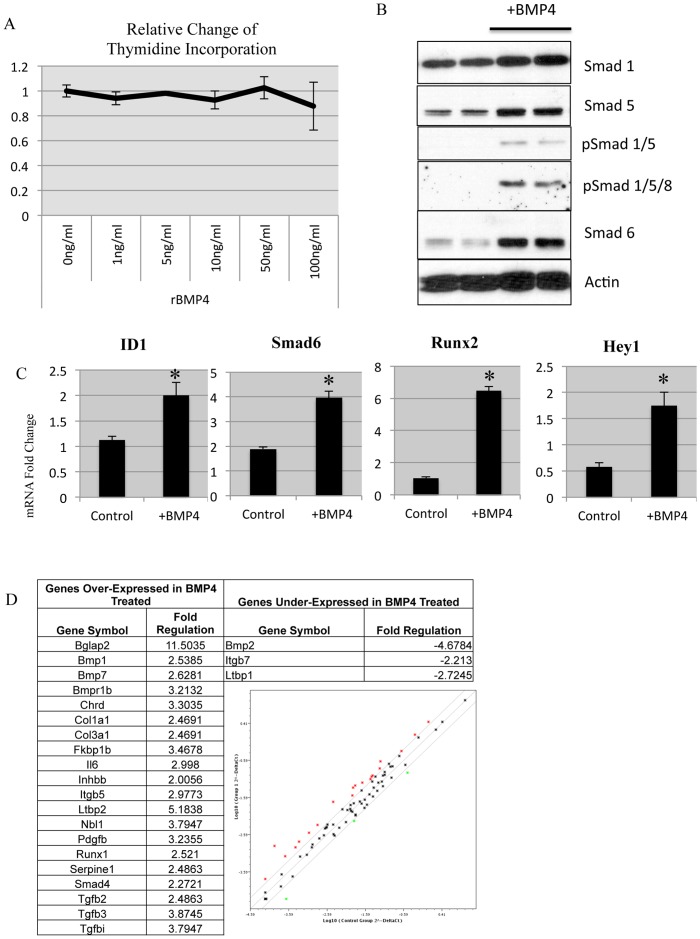
Response of mouse mammary fibroblasts to BMP4 stimulation. A) Increasing concentrations of recombinant mouse BMP4 added for 24 hours to measure proliferation via H^3^-Thymidine uptake. B) Western blot from mouse mammary fibroblasts treated for 24 hours with 100 ng/ml recombinant BMP4. Antibodies are specific for BMP mediated signaling, where pSmad is indicative of phosphorylated Smads. C) Real time PCR of canonical BMP targets measured after 24 hours of 100 ng/ml of recombinant BMP4. Samples are normalized relative to GAPDH mRNA expression and displayed as fold change. * Indicates statistical significance with a p-value <.01 from a student t-test. Error bars indicate standard deviation. D) mRNA from mammary fibroblasts treated with or without BMP4 (from B) were compared with RT-PCR array specific for TGFβ family members. A representative list of significantly altered genes from TGFβ focused array.

Bone Morphogenetic Proteins (BMP) were previously shown to regulate cell cycle progression in various breast cancer cell types [22]. We investigated the ability of recombinant BMP4 to induce growth arrest by titering increasing amounts of BMP4 up to 100 ng/ml and did not observe a reduction in proliferation as measure by uptake of H^3^-Thymidine (Fig. 1A). Active canonical BMP signaling is indicated by the presence of phosphorylation of Smads1/5/8 and upon stimulation of mouse mammary fibroblasts we could detect this effect 24 hours after addition of 100 ng/ml of BMP4 (Fig. 1B). Furthermore, BMP stimulation induces the expression of the inhibitory Smad6, which was also detected (Fig. 1B).

To further validate that mouse mammary fibroblasts were responding to BMP4 stimulation, real time-PCR (RT-PCR) was performed to measure mRNA changes in response to 24 hours of 100 ng/ml BMP4 treatment. We found that BMP response genes were all modestly induced (Fig. 1C). Smad7 is routinely observed to be induced by BMP4 stimulation [2], [20], yet is also commonly associated as the inhibitory Smad for TGFβ signaling. In order to identify any additional TGFβ superfamily changes resulting from BMP4 stimulation, we performed a TGFβ superfamily focused RT-PCR array, this demonstrated unique alterations in TGFβ signaling components (Fig. 1D).

Interestingly, within the TGFβ superfamily genes identified as altered after 24 hours of BMP4 treatment, we observed several distinct phenomena. First, we observed that BMP4 increased BMP7 while reducing BMP2 mRNA, which may suggest distinct roles for these ligands. Furthermore, we found that Col1a1 and Col3a1 were induced. Given the role of collagen production by fibroblasts, this suggested that BMP4 was altering the extracellular matrix (ECM). IL-6, a TGFβ target of inflammation, was also induced by BMP4 treatment. This demonstrates a role for BMP4-inducing inflammation via mammary gland fibroblasts. TGFβ is known to induce morphological changes in fibroblasts and activating them to myofiboblasts [23]. We also examined morphological changes and fibroblast markers and did not detect significant differences with BMP stimulation. All cells were also consistently positive for fibroblast markers (Fig. S1).

### BMP Induction of Secreted Factors

**Figure 2:**
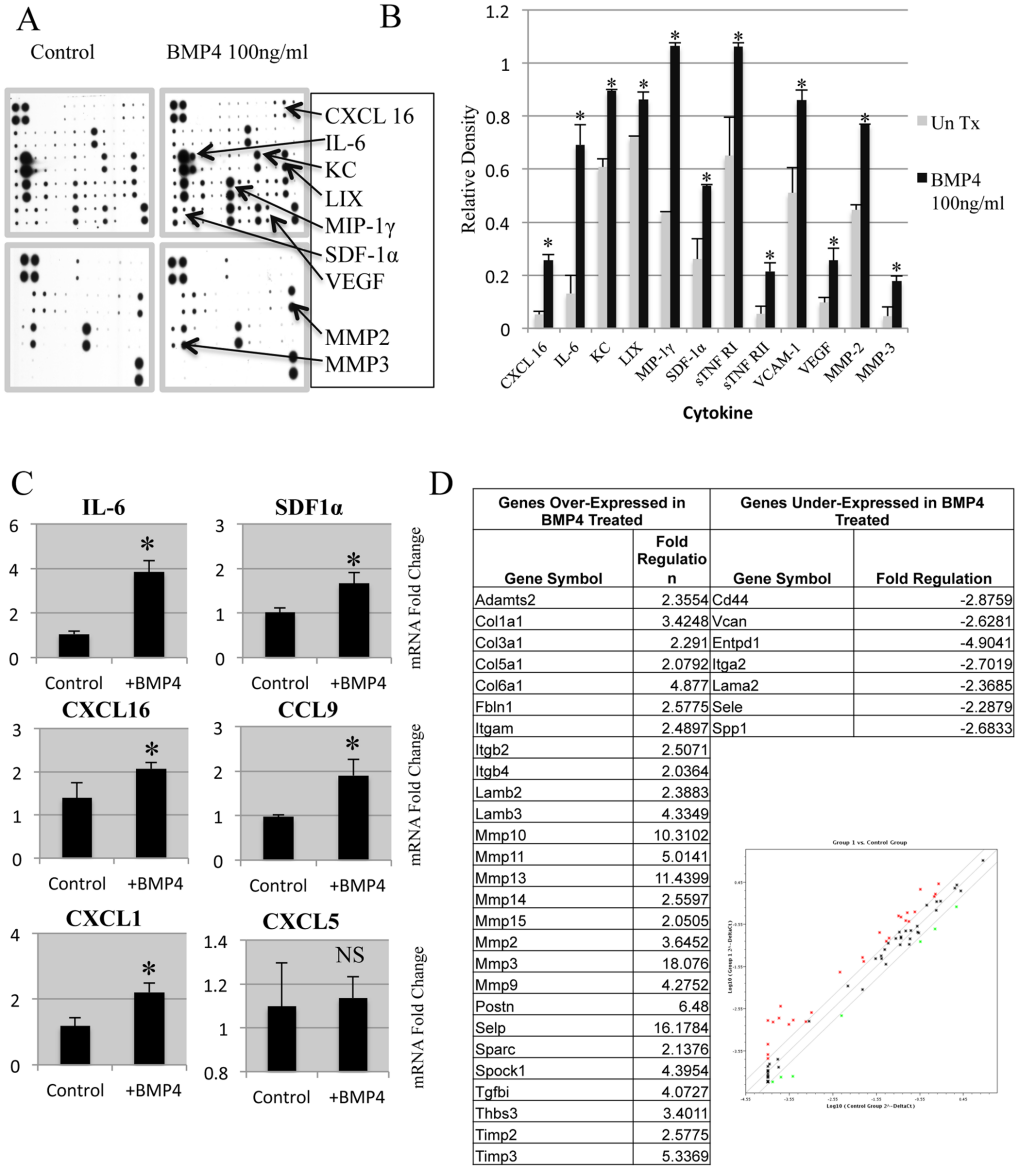
BMP4 treatment of mammary fibroblasts increases pro-tumorigenic cytokines and proteases. A) Mammary Fibroblasts were treated for 24 hours with 100 ng/ml of BMP4 and conditioned medium was collected and incubated with membrane bound antibody cytokine arrays. Arrays were scanned as TIFF images. B) Images were normalized to control spots within the arrays and quantified using NIH ImageJ software to determine relative intensity. C) Real time PCR validation of selected identified secreted proteins revealed molecules with known pro-tumorigenic functions including cytokines but also proteases such as MMP2 and MMP3 that facilitate tumor invasion and metastasis. Samples are normalized relative to GAPDH mRNA expression and displayed as fold change. * Indicates statistical significance with a p-value <.01 from a students t-test. D) Real time PCR focused array for specific ECM components further confirmed increased MMP transcription by BMP4 stimulation of mouse mammary fibroblasts. Error bars indicate standard deviation.

Our original hypothesis to test the tumor suppressive or promoting nature of BMP on fibroblasts was bolstered by the induction of IL-6 (Fig. 1D). We followed this by analyzing conditioned medium from BMP4 stimulated fibroblasts and identifying secreted factors by cytokine antibody array (Fig. 2A). Treatment of mouse mammary fibroblasts with BMP4 resulted in the increase of several soluble factors such as CXCL16, SDF1α, VEGF, MMP’s 2 &3, and importantly IL-6 (Fig. 2A&B). We confirmed several of these secreted factors by RT-PCR, which indicated a potent induction of several of these pro-tumorigenic factors including IL-6, SDF1α, CXCL16, CCL9, and CXCL1, but not CXCL5 (Fig 2C).

In order to examine the role of BMP signaling in mammary fibroblasts on expression of ECM components and their modifiers, a focused RT-PCR array for ECM and MMPs was performed. We found that many MMPs were strongly induced by a 24 hr stimulus of BMP4 at a concentration of 100ng/ml (Fig. 2D). Specifically, we discovered that BMP4 stimulation of fibroblasts was sufficient to induce the transcription of the mRNA of many MMPs. These MMPs (2,3,9,10,11,13,14,15) along with TIMPs suggest that mammary fibroblasts respond to BMP stimulation to enhance proteolytic degradation of the surrounding ECM (Fig. 2D). This elevated matrix remodeling can be indicative of a microenvironment that is supportive of enhanced tumor invasion.

### BMP Stimulation of both Mouse and Human Fibroblasts Enhances Tumor Cell Invasion and can be Inhibited by DMH1, a BMP Receptor Kinase Inhibitor

**Figure 3:**
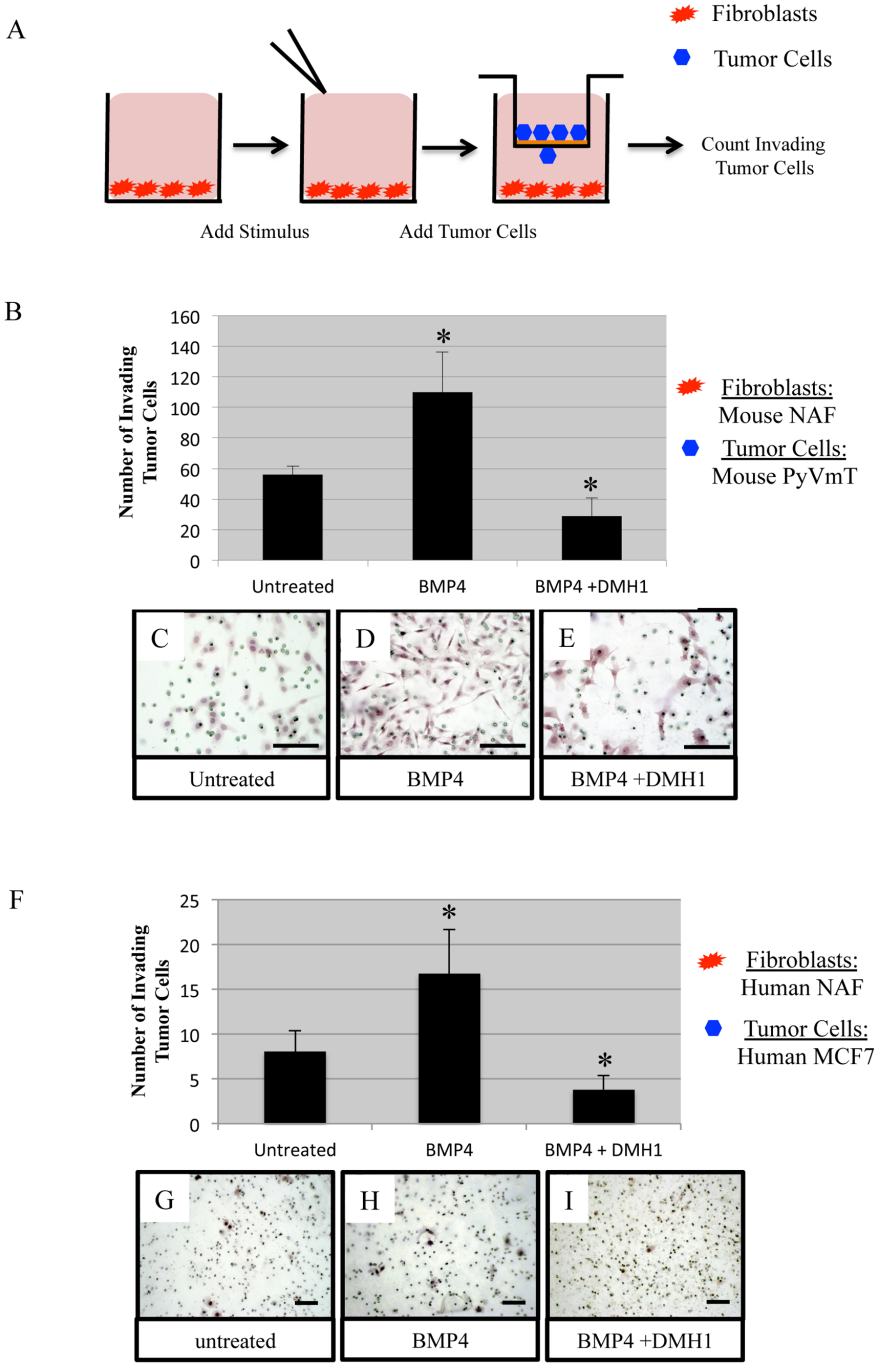
BMP4 Stimulation of Mammary fibroblasts promotes tumor invasion. A) Mammary fibroblasts were conditioned in untreated 10% FBS DMEM media, 100 ng/ml BMP4, and 20 µM DMH1 in a 24-well culture plate. B) Mouse mammary fibroblasts derived from a healthy C57BL6 female were seeded at 5×10^4^. After 24 hours fibroblasts were treated for 24 hours with BMP4 and/or DMH1. Fresh 10% FBS DMEM media replaced treatments while 2.5×10^4^ MMTV-PyVmT tumor cells were added to BD-Matrigel® inserts and allowed to invade for 18 hours. Cells were counted at a 20× magnification and the average of five images for each triplicate was calculated. Representative images of tumor cells that have invaded through 8 µm pores onto the underlying side are shown for untreated fibroblasts (C) and BMP4 stimulated fibroblasts (D) and BMP4 with the BMP receptor kinase inhibitor DMH1 (E). F) Human fibroblasts derived from reduction mammoplasty tissue (NAF) were seeded at 5×10^4^. After 48 hours fibroblasts were treated for 24 hours with BMP4 and/or DMH1. Fresh 10% FBS DMEM media replaced treatments while 1×10^5^ MCF7 tumor cells were added to BD-Matrigel inserts and allowed to invade for 48 hours. Cells were counted at a 10× magnification and the average of five images for each triplicate was calculated. Representative images of tumor cells that have invaded through 8 µm pores onto the underlying side are shown for untreated fibroblasts (G) and BMP4 stimulated fibroblasts (H) and BMP4 with the BMP receptor kinase inhibitor DMH1 (I). Scale bars indicate 50 µM. * Indicates statistical significance of a p-value <.01 by performing a 2-tailed students T-test by comparing data to the untreated samples. Error bars indicate standard deviation.

To determine the functional significance of BMP-treatment of fibroblasts on carcinoma cell invasion, we designed an experiment where we culture normal mouse mammary fibroblasts (NAFs) in a 24-well culture dish. Sub-confluent fibroblasts were treated for 24 hours with BMP4 at 100 ng/ml. We additionally treated the fibroblasts with a combination of 100 ng/ml BMP4 with the selective and specific BMP receptor kinase antagonist DMH1. DMH1 is a potent inhibitor of BMP signaling [24], [25]. Following treatment, media containing BMP4 and/or DMH1 was removed, and fresh media without BMP4 or DMH1 was added to the fibroblasts. Next, 8 µM pore invasion chambers coated with BD Matrigel containing 2.5×10^4^ mouse breast carcinoma tumor cells (MMTV.PyVmT derived) were allowed to invade and cross through the pores for 18 hours and then quantified (Fig 3A).

Hematoxylin stained tumor cells that had invaded through 8 µM pores were photographed and counted to determine the number of tumor cells invaded. BMP4 stimulated fibroblasts significantly increased the number of tumor cells invaded through BD Matrigel, while inhibition of BMP signaling via DMH1 treatment statistically significantly reduced the number of invaded tumor cells (Fig. 3B). Morphological changes of the cells were also evident; fibroblasts stimulated by BMP4 altered tumor cells to appear more spindle-like and dysplastic when compared with controls and DMH1 treated fibroblasts (Fig. 3C–E).

Mouse and human secreted cytokines and ECM modulators are known to have distinct differences [26]. To determine whether human NAFs behave in the same way as mouse NAFs we repeated the invasion assay with human fibroblasts and tumor cells. Human fibroblasts derived from reduction mammoplasty tissue were treated for 24 hours with BMP4 and/or DMH1 and then fresh media was added to the fibroblasts as performed above. MCF7 cells are known to be poorly invasive and chosen to determine whether BMP stimulation of fibroblasts could enhance invasion as opposed to attempting to enhance highly invasive tumor cells that possess cell-autonomous invasive properties. MCF7 cells were much less invasive than PyVmT expressing mouse carcinoma cells and required four times as many cells seeded (1×10^5^) and 48 hours to invade through the Matrigel matrix. Similar to the mouse experiments BMP4 stimulation increased MCF7 tumor cell invasion and was reduced with the BMP receptor inhibitor DMH1 (Fig. 3F). However, unlike mouse carcinoma cells, MCF7 cells did not reveal any distinct morphological changes following invasion (Fig 3G–I).

While DMH1 has been demonstrated to be a specific and selective inhibitor of BMP signaling [25], we wanted to ensure that the effects were not limited by less fibroblast survival. We assayed an increasing titer amount and did not observe changes in cell number and viability (Fig. S2A). We also did not identify any significant changes in apoptosis of the fibroblasts relative to their DMSO controls (Fig. S2B).

### Human Carcinoma Associated Mammary Fibroblasts Display Unique Changes in BMP Signaling Components Compared to Normal Human Mammary Fibroblasts

**Figure 4:**
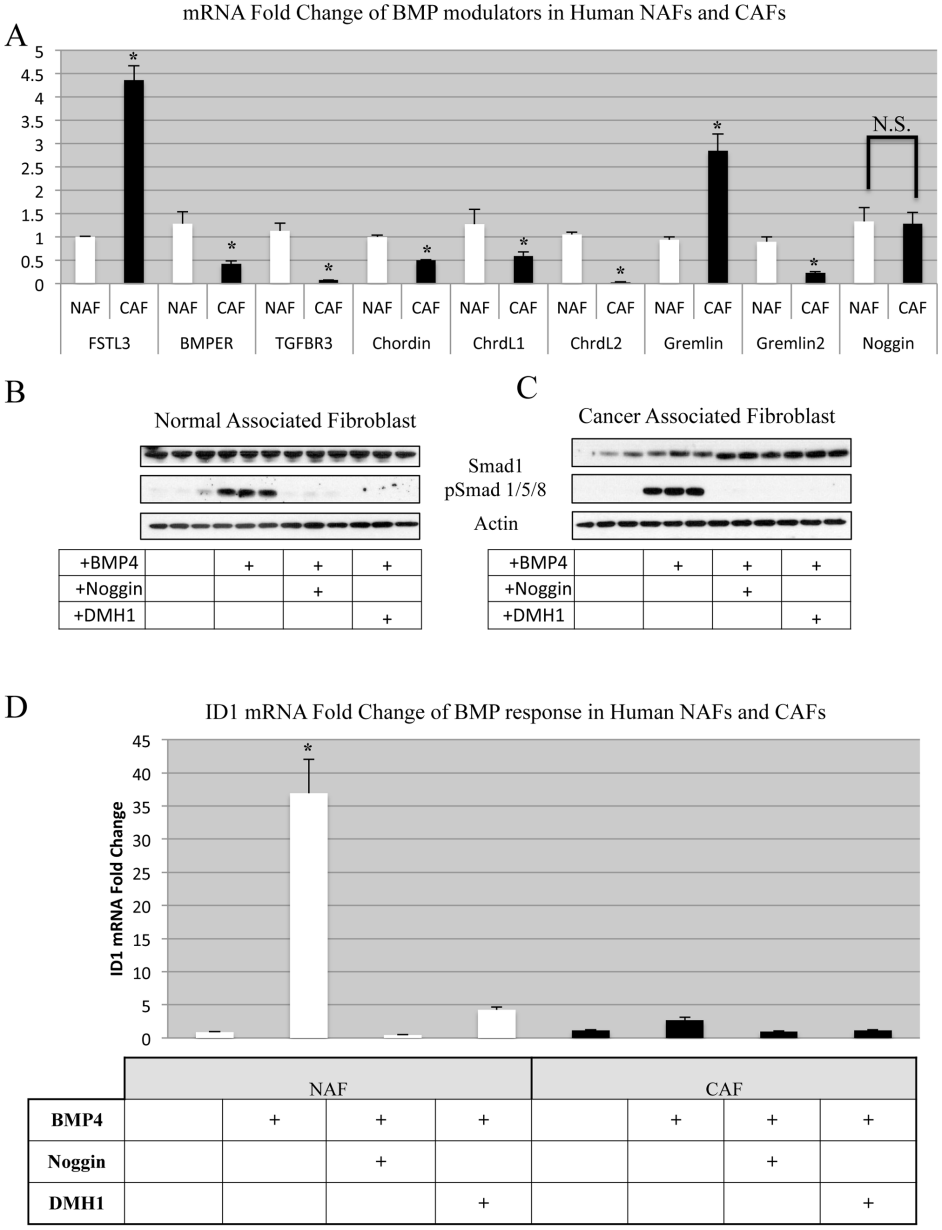
BMP signaling is altered in Human Breast CAF compared to NAF. Normal Associated Fibroblasts (NAF) and Carcinoma Associated Fibroblasts (CAF) were derived from human breast tissue RNA was isolated an analyzed by SYBR RT-PCR. A) RT-PCR was performed for known modulators of the BMP pathway. Samples are normalized relative to GAPDH mRNA expression and displayed as fold change. * Indicates statistical significance with a p-value <.01 from a student t-test. N.S. indicates no significance between two groups. B&C) Western blot for BMP response revealed active BMP signaling in NAF and CAF. Cells were serum starved overnight and treated in serum free medium with recombinant human BMP4 (100 ng/ml), Noggin (1 ug/ml) and DMH1 (20 µM). D) RT-PCR for BMP response canonical target gene *id1* after 24 hours of treatment with BMP4 (100 ng/ml), Noggin (1 ug/ml) and DMH1 (20 µM) demonstrated unique transcriptional response between NAF and CAF. Error bars indicate standard deviation.

BMP signaling activity can be inhibited by more than 20 secreted soluble antagonists [2]. Furthermore, there exist co-receptors of TGFβ and BMP signaling that are known to modulate ligand stimulation of receptors. We performed RT-PCR for expression of these modulators and found significant differences in fibroblasts isolated from normal human breast tissue (NAF), compared with fibroblasts isolated from breast cancer (CAF) (Fig. 4A). Noggin, a chief soluble antagonist of BMP signaling, (which we used to antagonize BMP stimulation *in vitro*) was not significantly altered and was not expressed at high levels. However, many modulators were significantly absent in CAF cells such as Chordin molecules, while two molecules that significantly increased were Gremlin (GREM1) and Follistatin-like 3 (FSTL3) (Fig. 4A). Additionally, co-receptors of BMP signaling TGFβR3 and BMPER were significantly lost, demonstrating a switch in the pathway response to BMP signaling.

We next sought to establish by protein analysis what functional competency these cells (NAFs and CAFs) served for BMP signaling. NAF cells when stimulated with BMP4 showed a marked induction of pSmad1/5/8 (Fig. 4B). Inhibition of BMP4 was achieved with either Noggin or DMH1 treatment of NAFs (Fig. 4B), and both inhibited BMP4 stimulation. We next examined how CAF cells responded to BMP4, and found that BMP4 treatment of these cells phosphorylated Smads 1,5 and 8. This phosphorylation indicated that both NAF and CAF cells had intact canonical BMP signaling. Additionally, BMP inhibition either by Noggin or DMH1 was sufficient to block the phosphorylation of Smads 1,5 and 8 (Fig. 4B&C).

We proceeded to correlate the observed protein response to the transcriptional response to BMP4 by RT-PCR and observed that BMP signaling could be activated in NAF cells, yet CAF cells appeared unresponsive (Fig. 4D). We observed that NAF cells had a robust increase in the canonical BMP target gene ID1, which could be attenuated with the addition of the inhibitors, Noggin or DMH1 (Fig. 4D,). We also observed that Smad6 mRNA was not altered significantly in CAF cells in response to any BMP4 stimulation or inhibition (data not shown). We performed additional experiments in serum free and full serum media and did not see any difference in response (data not shown). As we observed for protein, ID1 was modestly induced by BMP4 treatment in CAF cells and inhibited with Noggin or DMH1 (Fig. 4D). However, these responses were markedly less than NAF response for ID1 than in CAF cells (Fig. 4D).

### BMP Alterations of IL-6 and MMP-3 is Different in Human NAFs Compared with CAFs

**Figure 5:**
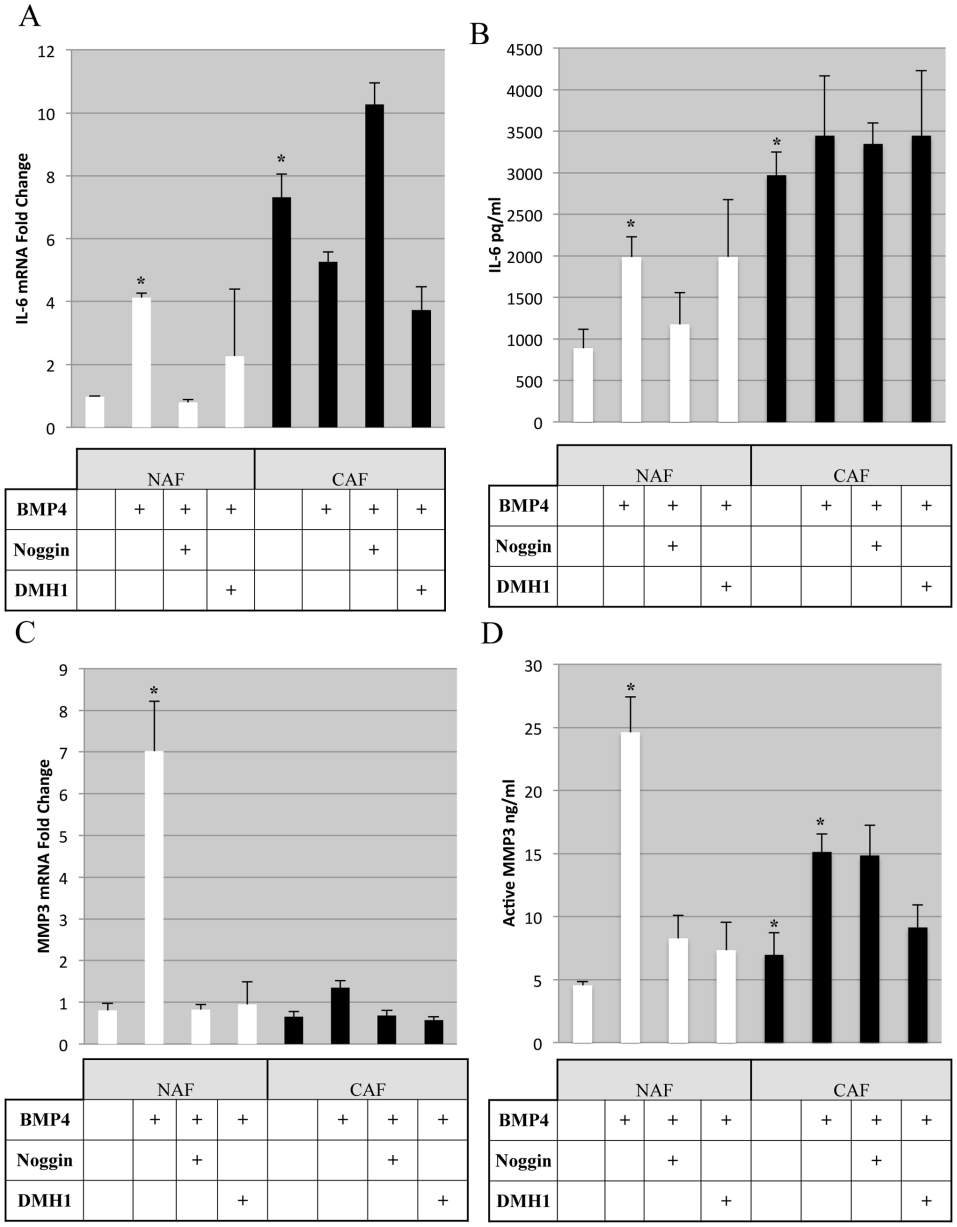
Human NAF and CAF reveal distinct BMP stimulation of pro-tumorigenic factors IL-6 and MMP-3. A) RT-PCR from RNA harvested from Human NAF and CAF cells undergoing treatments indicated for 24 hours. Cells were serum starved overnight and treated in serum free medium. B) ELISA for IL-6 protein levels from conditioned medium following 24 hours stimulation in serum free medium comparing NAF vs. CAF response to BMP signaling. C) RT-PCR from the same cells as (A) analyzed for relative human MMP-3 mRNA. D) Active human MMP-3 protein levels from the same conditioned medium as (B) were assayed with ELISA specific antibodies for total and active MMP-3. * Indicates statistical significance performing a 2-tailed Students T-test comparing the untreated sample, error bars indicate standard deviation among biological triplicates. BMP4 was added at 100 ng/ml for 24 hours, and was included with Noggin (1 ug/ml) or DMH1 (20 uM) treatments.

We observed that BMP stimulation of mouse mammary fibroblasts can produce pro-tumorigenic paracrine factors, and wanted to investigate how human NAF and CAF cells behaved with respect to these factors. We first analyzed by RT-PCR mRNA IL-6 levels and found that CAF cells were expressing significantly higher levels of IL-6. This increase was not lowered to untreated NAF levels when either stimulated with BMP4, or inhibited with Noggin or DMH1 (Fig. 5A). Importantly, we measured secreted IL-6 in conditioned medium from NAF and CAF cells and found that BMP4 treatment increased secretion of IL-6 protein in NAF cells. However, in CAF cells there existed much higher levels of IL-6 protein expression than even BMP4 could induce in NAFs, and the CAF levels were not decreased with BMP inhibition (Fig. 5B). Interestingly, while DMH1 had been effective at inhibiting BMP targets Smad6 and ID1 (Fig. 4D), it was not as effective as Noggin in reducing IL-6 mRNA levels (Fig. 5A) and IL-6 protein expression (Fig. 5B).

We next determined whether MMP-3 expression was altered in NAFs and CAFs and found that MMP-3 mRNA was not significantly different in NAFs and CAFs, but was strongly induced by BMP4 treatment in NAFs and only modestly induced in CAFs (Fig. 5C). Interestingly, unlike IL-6, MMP-3 mRNA induction was inhibited with both Noggin and DMH1 treatment in NAFs and was largely unaffected in CAFs (Fig. 5C). MMP-3 mRNA does not directly indicate activity of the protease, so we investigated not only the total MMP-3 secreted, but the active MMP-3 levels in NAF and CAF cells. An ELISA assay for active MMP-3 showed that CAF cells had higher levels than NAFs, and could be increased in both NAF and CAF cells by BMP4 treatment (Fig. 5D). Active MMP-3 was reduced by both Noggin and DMH1 inhibition in NAFs (Fig. 5C). However, treatment of CAFs with BMP4 only minimally increased active MMP-3 when compared with levels in NAFs, and Noggin treatment had little effect. Interestingly, DMH1 inhibition was sufficient to reduce active MMP3 in CAFs, albeit not completely to the untreated levels in CAFs or NAFs (Fig. 5D).

## Discussion

Previously, we reported that the loss of BMP signaling in the mammary epithelium accelerated mammary carcinoma progression [16]. Here, we show that active BMP signaling is sufficient to enhance carcinoma cell invasion via stimulation of stromal fibroblasts. These observations in fibroblasts are consistent with previous reports where BMP2 and BMP7 were shown to support tumors acting through fibroblasts derived from prostate adenocarcinomas [12], [18]. This coupled to the reports that BMP ligands are increased in breast cancer [18] as well as other cancers [27] may help explain the function of these ligands in breast cancer. Loss of BMP signaling in the epithelium and diversion to the stroma can therefore serve as a switch to BMP mediated promotion of tumorigenesis. Interestingly, it has been shown that while BMP can induce growth arrest in human breast cancer cells, they simultaneously promote enhanced migration [22]. Recently, a study investigating a stromal gene signature in DCIS and IDC samples uncovered a signature of genes that are changed in this transition. Within this signature it was found that Grem1 (a BMP antagonist) was upregulated in DCIS and IDC [28]. Interestingly, Grem1 has been shown to be widely upregulated in the underlying stroma of cutaneous basal cell carcinomas [29] when compared to normal stroma in skin. Our data also demonstrated an increase in Grem1 in CAFs compared with NAFs (Fig. 4A) yet was accompanied by the loss of expression of many other soluble BMP antagonists. It remains to be seen how this unique antagonist functions in the context of many ligands and alternate soluble antagonists and co-receptors. Further identification of ligands and modulators may be needed to fully understand the mechanism of BMP and other pathway activity.

BMP4 induction of IL-6 could enhance the inflammatory tumor microenvironment (Fig.1, 2 & 5). IL-6 is a master regulator of inflammation and correlates with poor outcome and survival in breast cancer [30], [31]. Specifically, serum levels of IL-6 independent of activity, predict poor outcome in breast cancer [30], [32]. It has been shown that fibroblasts derived from metastatic breast cancers have IL-6 dependent growth and invasion [33]. While we demonstrated BMP4 induction of IL-6, there were certainly other inflammatory molecules induced by BMP treatment of fibroblasts (Fig. 2). BMP responses in dermal fibroblast and keratinocytes were previously reported in a microarray profiling that revealed the distinct responses from the epithelium and the stroma [20]. In this study it was interesting to note that many pro-tumorigenic factors were upregulated specifically in fibroblasts, and not in the epithelium derived keratinocytes [20]. BMP mediated inflammation has also been also demonstrated for additional cells that reside in the tumor microenvironment. BMP6 stimulation of macrophages is sufficient to induce IL-1β expression, which can enhance inflammation [34]. Furthermore, it has been shown that BMP6 secreted from prostate tumor cells acts directly upon macrophages to stimulate secretion of IL-6, which ultimately results in neuroendocrine differentiation of the tumor [34]. Taken together, these findings demonstrate a pro-inflammatory role for BMP activity in the stroma of tumors.

BMP4 stimulation of fibroblasts was sufficient to increase the expression of many ECM component and regulators (Fig. 2). The ECM has been shown to be a critical regulator of breast cancer and the progression to metastasis [11]. MMPs, specifically MMP-3, have been shown to promote mammary tumor progression [35]. MMP-3 has also been shown to facilitate breast cancer metastasis to the brain, which was blocked by MMP inhibition [36]. Others have shown that BMPs can inhibit growth of epithelial cells [22], however this did not occur with mammary fibroblasts (Fig. 1A). We briefly stimulated fibroblasts with BMP4 and then added tumor cells to Matrigel covered invasion chambers and found that fibroblasts could not only increase invasion through the matrix, but be inhibited pharmacologically with DMH1. We were able to demonstrate that BMP4 stimulation of both mouse and human fibroblasts was sufficient to enhance mouse and human breast carcinoma invasion (Fig. 3). The use of DMH1 to inhibit BMP signaling not only reduced fibroblast stimulated by BMP4 but also inhibited invasion below un-stimulated fibroblasts in both mouse and human cells (Fig. 3). DMH1 is a selective and specific kinase inhibitor for BMP type 1 receptors [21]. Recently, it has been shown that inhibition of prostate tumors with another kinase inhibitor LDN-193189 slowed tumor growth, but also inhibited the osteogenic program that these tumors undergo [37]. Treatment of prostate cancer’s osteolytic bone metastasis with Noggin has also shown to be a potentially successful strategy [38]. More recently, LDN-193189 was used to treat a mouse model of breast cancer and found to be effective directly at limiting tumorigenicity [39]. It remains unclear whether there were effects on the tumor microenvironment or the tumor cells with LDN-193189 administration.

While dual targeting of IL-6 and MMP3 may provide unique challenges, BMP represents a new pathway of signaling molecules that may be targeted for therapeutic intervention. Our studies and others showing such disparate responses by BMP stimulation in epithelial cells and fibroblasts suggest caution going forward for the use of BMPs as targets in cancer. Such has been the case with TGFβ targeted therapies where both agonists and antagonists have been considered. Recently, treatment with a Dorsomorphin analog LDN-193189, which can inhibit BMP signaling similarly to DMH1, has been used successfully to limit prostate tumor growth, and bone metastases [37]. This supports work demonstrating that BMP2 stimulated angiogenesis in lung tumors and could be ameliorated by treatment with LDN-193189 [40]. Interestingly, DMH1 is a more selective and specific inhibitor of BMP signaling than LDN-193189, which is known to antagonize VEGF and TGFβ signaling as well [25]. This is not to say that selectivity or specificity is required for effective therapy and that inhibition of these other pathways may be suitable. Further work *in vivo* in various models coupled with genetic deletion of BMP signaling is specific stromal subtypes will be useful to determine the role of BMP in the tumor microenvironment. Interestingly, it has recently been shown that BMP4 is associated with lymphatics that have less metastatic ability, suggesting that inhibition could be harmful. On the other hand, it was recently demonstrated that BMP signaling was responsible for the growth of new lymphatics independent of arterial growth, which led the authors to speculate inhibition could be used in cancer to prevent lymphatic spread into tumors and dissemination into draining lymph nodes [41]. Methods of modulating BMP signaling *in vivo* have been currently limited to systemic treatments, but perhaps with the recent advances in cell type specific targeting [42], [43], their use can be developed against specific cell populations that support tumor progression.

These findings suggest that the BMP pathway represents a useful target in the tumor microenvironment in breast cancer. Inhibition of BMP signaling in fibroblasts may provide a successful adjuvant to current therapies, given that fibroblasts are usually exempt from mutations found within the tumor [44], [45]. Limiting the ability of fibroblasts to increase inflammation and ECM degradation via BMP inhibition should be further investigated as a potential therapy aimed at the tumor microenvironment. Even the most difficult to treat tumors, which correlate with higher inflammation and tumor invasion, would potentially be suitable for BMP inhibition. Further elucidation of the BMP pathway in tumor cells and the diverse cells of the tumor microenvironment is warranted.

## Supporting Information

Figure S1 **BMP4 treatment does alter markers of fibroblasts.** Differentiation markers that are typical of fibroblasts are stained with antibodies listed (green) and counterstained with DAPI (blue) to highlight the nuclei of all cells. Scale bars indicate 100 µM.
(TIF)

Figure S2 **DMH1 is not Toxic to Mammary Fibroblasts.** Mammary Fibroblasts were treated with varying amounts of DMH1 in 10% FCS DMEM for 24 hours in a 96-well plate. An equal amount of DMSO was added as a control since DMH1 was diluted in DMSO. After 24 hours, using the MultiTox-Glo Multiplex Cytotoxicity Assay, fluorescent readings were taken to assess cell viability. Fluorescence was measured in relative fluorescence units (RFUs). Results indicate that high concentrations of DMH1 did not significantly reduce cell viability. Next, luminescence was assessed in relative luminescence units (RLU) to measure cell death. Results show that apoptosis only increased slightly with DHM1 concentrations of 20 uM and higher. DMSO controls show that DMSO did not significantly alter results.
(TIF)

Table S1 **Primer sequences.** Primers were designed via NCBI:GENE-http://www.ncbi.nlm.nih.gov/gene/. Where the correct gene was identified (mouse or human) and selected for the correct mRNA transcript in NCBI:Nucleotide. Following the “pick primers” option under “analyze sequence” menu, PCR product size was limited to 120 bp and alternate spliceform was selected for search. When Exon junction spanning primers were available they were chosen, otherwise the first primer pair was selected.
(TIF)
