# Supplementary figures and images for: Bone Morphogenetic Proteins Stimulate Mammary Fibroblasts to Promote Mammary Carcinoma Cell Invasion
Source: PLoS One. 2013 Jun 28;8(6):e67533. doi: 10.1371/journal.pone.0067533 (PMC3695869; doi:10.1371/journal.pone.0067533)

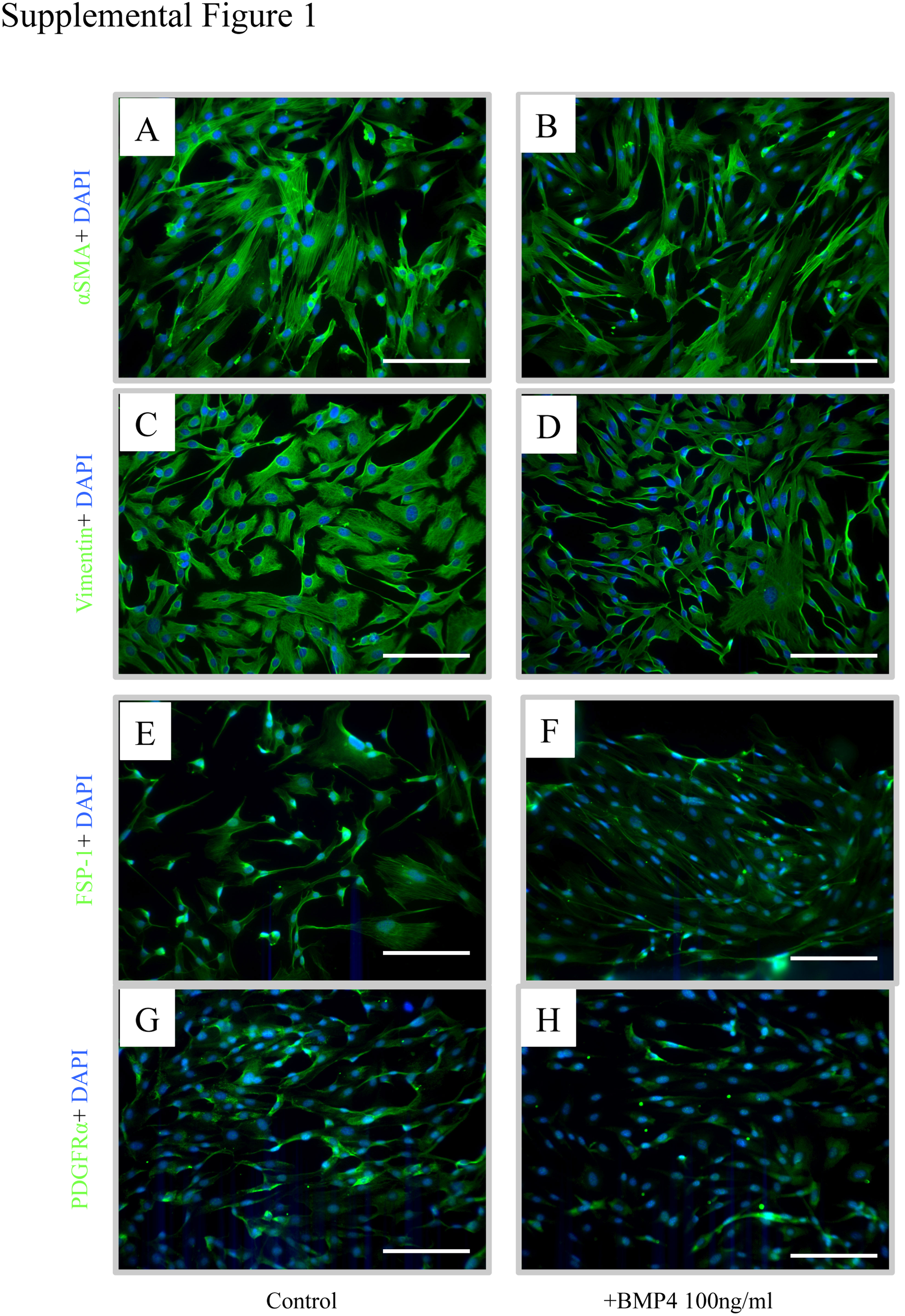

Supplement: Figure S1 — BMP4 treatment does alter markers of fibroblasts. Differentiation markers that are typical of fibroblasts are stained with antibodies listed (green) and counterstained with DAPI (blue) to highlight the nuclei of all cells. Scale bars indicate 100 µM. (TIF) [file pone.0067533.s001.tif]

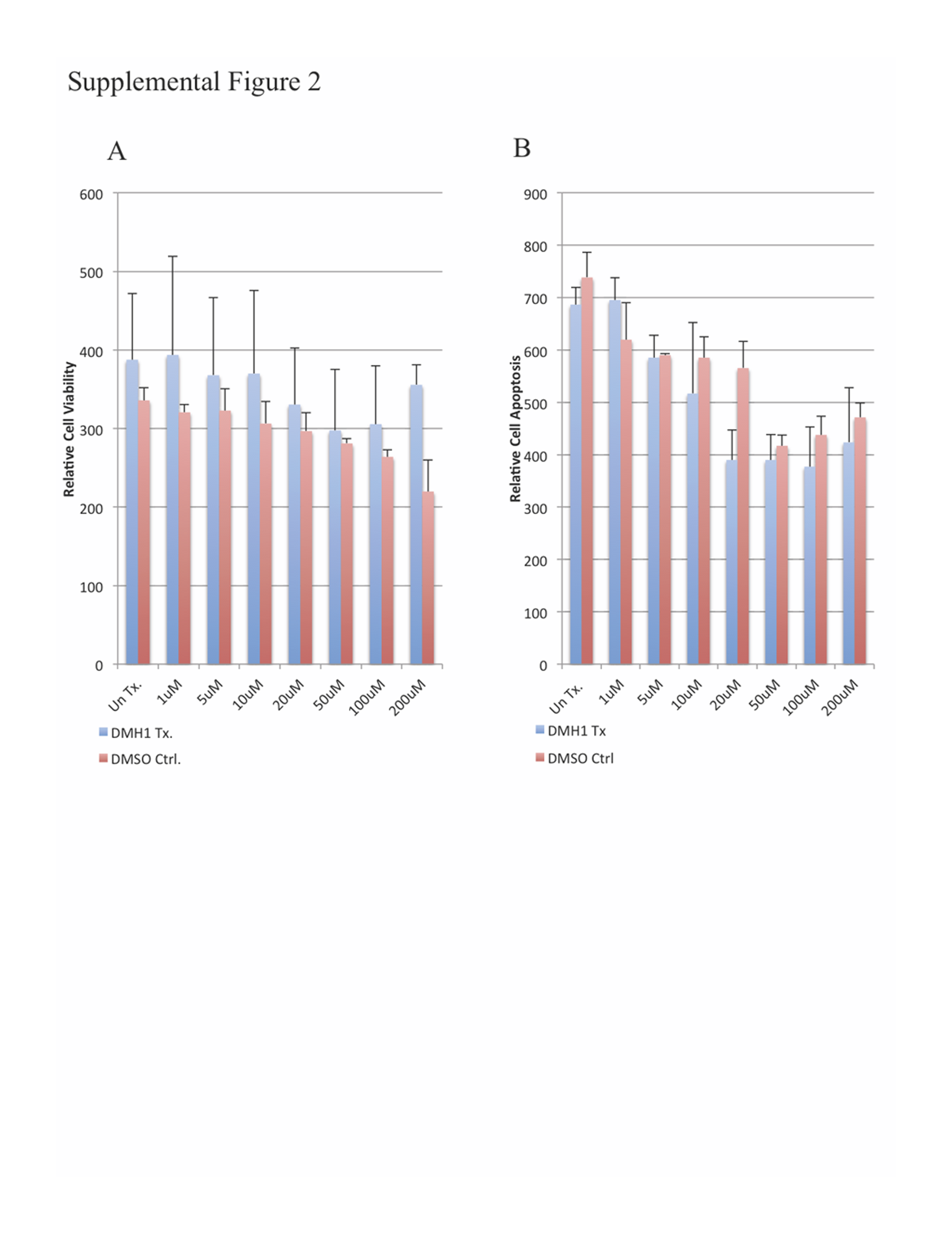

Supplement: Figure S2 — DMH1 is not Toxic to Mammary Fibroblasts. Mammary Fibroblasts were treated with varying amounts of DMH1 in 10% FCS DMEM for 24 hours in a 96-well plate. An equal amount of DMSO was added as a control since DMH1 was diluted in DMSO. After 24 hours, using the MultiTox-Glo Multiplex Cytotoxicity Assay, fluorescent readings were taken to assess cell viability. Fluorescence was measured in relative fluorescence units (RFUs). Results indicate that high concentrations of DMH1 did not significantly reduce cell viability. Next, luminescence was assessed in relative luminescence units (RLU) to measure cell death. Results show that apoptosis only increased slightly with DHM1 concentrations of 20 uM and higher. DMSO controls show that DMSO did not significantly alter results. (TIF) [file pone.0067533.s002.tif]

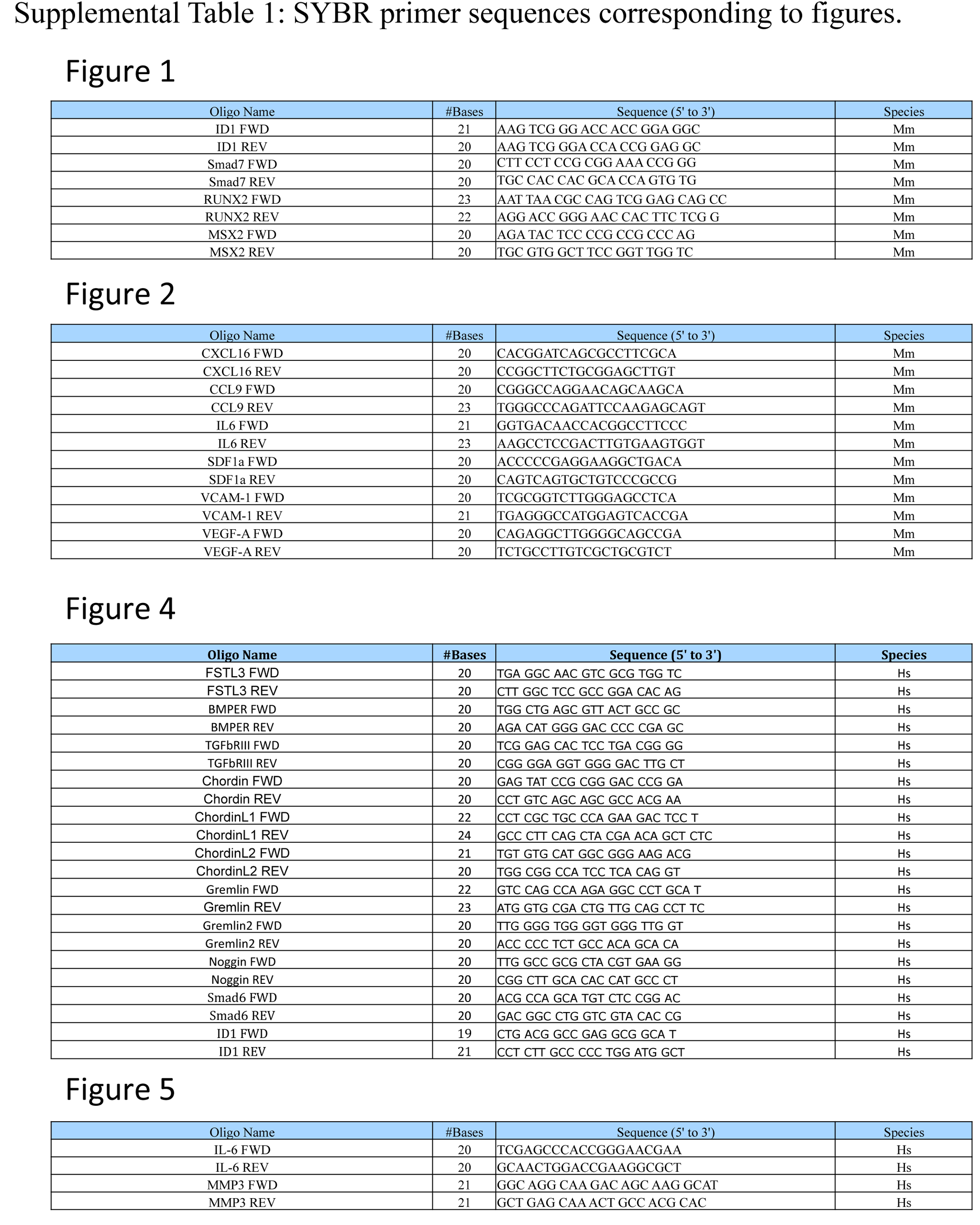

Supplement: Table S1 — Primer sequences. Primers were designed via NCBI:GENE-http://www.ncbi.nlm.nih.gov/gene/. Where the correct gene was identified (mouse or human) and selected for the correct mRNA transcript in NCBI:Nucleotide. Following the “pick primers” option under “analyze sequence” menu, PCR product size was limited to 120 bp and alternate spliceform was selected for search. When Exon junction spanning primers were available they were chosen, otherwise the first primer pair was selected. (TIF) [file pone.0067533.s003.tif]
